# PICOLINIC ACID, A TRYPTOPHAN METABOLITE, DOESN’T AFFECT BONE MINERAL DENSITY BUT UPREGULATES LIPID STORAGE GENES

**DOI:** 10.1093/geroni/igz038.375

**Published:** 2019-11-08

**Authors:** Carlos Isales, Meghan McGee-Lawrence, Mark Hamrick, William D Hill, Wendy Bollag, Ke-Hong Ding, Xing-ming Shi, Eileen Kennedy

**Affiliations:** 1 Augusta University, Augusta, Georgia, United States; 2 Medical University of South Carolina, Charleston, South Carolina, United States; 3 University of Georgia, Athens, Georgia, United States

## Abstract

Tryptophan is an essential amino-acid broken down initially to kynurenine (kyn), an immunomodulatory metabolite that we have previously shown to promote bone loss. Kyn levels increase with aging and have also been associated with neurodegenerative disorders. Additional tryptophan metabolites include picolinic acid (PA); however, in contrast to kyn, PA is neuroprotective. Thus, we hypothesized that PA might be osteoprotective. In an IACUC-approved protocol, we fed PA to aged (23-month-old) C57BL/6 mice for eight weeks. In an effort to determine potential interactions of PA with dietary protein, we added PA to both a standard (18%) and a low protein diet (8%). The mice were divided into four groups: control (18% protein), +PA (700 ppm); low protein (8%), +PA (700 ppm). There was no difference in weight among the groups [36.1±4.1, 34.6±3.8, 32.8±3.2, 32.6±3.0 gm, (Means±SD, control vs +PA vs 8% vs +PA, p=ns; n=8-10/group). Mice were sacrificed and bones and stromal cells collected for analysis. We found that addition of PA to the diet had no impact on femoral BMD or BMC (BMD: 0.069±0.008 vs 0.075±0.007 vs 0.069±0.005 vs 0.070±007, p=ns). Addition of PA to the diet had no impact of % body fat as measured by DXA; however, stromal cells isolated from the PA-fed mice showed a significant increase in the expression of the lipid storage genes, Plin1 and Cidec. Thus, although PA is downstream of kyn, the kyn-induced detrimental effects on bone mass are no longer observed with PA but instead this kyn metabolite appears to impact energy balance.

